# Clinical Appearance of Oral Candida Infection and Therapeutic Strategies

**DOI:** 10.3389/fmicb.2015.01391

**Published:** 2015-12-17

**Authors:** Shankargouda Patil, Roopa S. Rao, Barnali Majumdar, Sukumaran Anil

**Affiliations:** ^1^Department of Oral Pathology and Microbiology, Faculty of Dental Sciences, M. S. Ramaiah University of Applied SciencesBangalore, India; ^2^Dental Biomaterials Research Chair, Dental Health Department, College of Applied Medical Sciences, King Saud UniversityRiyadh, Saudi Arabia

**Keywords:** antifungal therapy, *Candida*, NCAC species, oral candidosis, opportunistic infections

## Abstract

*Candida* species present both as commensals and opportunistic pathogens of the oral cavity. For decades, it has enthralled the clinicians to investigate its pathogenicity and to improvise newer therapeutic regimens based on the updated molecular research. Candida is readily isolated from the oral cavity, but simple carriage does not predictably result in development of an infection. Whether it remains as a commensal, or transmutes into a pathogen, is usually determined by pre-existing or associated variations in the host immune system. The candida infections may range from non-life threatening superficial mucocutaneous disorders to invasive disseminated disease involving multiple organs. In fact, with the increase in number of AIDS cases, there is a resurgence of less common forms of oral candida infections. The treatment after confirmation of the diagnosis should include recognizing and eliminating the underlying causes such as ill-fitting oral appliances, history of medications (antibiotics, corticosteroids, etc.), immunological and endocrine disorders, nutritional deficiency states and prolonged hospitalization. Treatment with appropriate topical antifungal agents such as amphotericin, nystatin, or miconazole usually resolves the symptoms of superficial infection. Occasionally, administration of systemic antifungal agents may be necessary in immunocompromised patients, the selection of which should be based upon history of recent azole exposure, a history of intolerance to an antifungal agent, the dominant *Candida* species and current susceptibility data.

## Introduction

The malady of thrush or candidiasis has been known to occur in people for over 2000 years. As mentioned by the famous Greek physician, Hippocrates in his findings, it commonly presents as superficial infections of the oral and vaginal mucosa. However, it was not until the mid-1800s that the documented research on pathogenesis of candidiasis were instigated. The principal yeast pathogen, *Candida albicans*, itself, was identified in the nineteenth century. In the early 1900s, *C. albicans* was found in the oral cavity of 54% of 2–6 weeks old and 46% of 1 year old infants and in 39% of 1–6 years old children, nonetheless several of them were rather healthy (Barnett, 2008). It was only later, that the subsequent studies revealed the normal oral carriage of *C. albicans* is 2.0–69.1% among the healthy adult population, depending upon the sampled population and technique (Scully et al., 1994).

In recent years, noteworthy escalation in pathogenic state of this commensal has been observed, as reflected in the increased incidence of the common and infrequent forms of candidiasis (Williams and Lewis, 2011). The probable explanations include changes in the practice of medicine like introduction of broad-spectrum antibiotics, immunosuppressive agents, transplantations, indwelling catheters, etc., and morbid conditions such as diabetes, severe malnutrition in children and AIDS (Lalla et al., 2013). Oral candidiasis is a significant source of morbidity, as it can cause chronic pain or discomfort upon mastication, limiting nutrition intake in the elderly or immunodeficient patients. There are multiple clinical presentations of oropharyngeal and esophageal candidiasis caused by *C. albicans*, either alone or in mixed infection (Sherman et al., 2002). Thus, with the above outlook, the present review comprehends the varied clinical manifestations and the current treatment strategies for this opportunistic pathogen.

## Epidemiology

Oral candidosis is frequent in the extremes of age (Akpan and Morgan, 2002). Approximately 5–7% of infants develop oral candidiasis. Its prevalence in AIDS patients is estimated to be 9–31% and close to 20% in the cancer patients (Lalla et al., 2013). The oral carriage of candida organisms is reported to be 30–45% in the general healthy adult population (Akpan and Morgan, 2002). The incidence of *C. albicans* in healthy and various health conditions is depicted in **Table 1**. The additional important species isolated from clinical infections include, *C. glabrata, C. guillierimondii, C. krusei, C. lusitaniae, C. parapsilosis, C. pseudotropicalis, C. stellatoidea*, and *C. tropicalis* (Crist et al., 1996). In recent years higher incidences of the above mentioned non- *C. albicans Candida* (NCAC) species have been also reported (Williams and Lewis, 2011).

**Table 1:** Oral carriage of *Calbicans albicans* in various subjects (Akpan and Morgan, 2002).

Subjects	Oral carriage of *C. albicans*
Neonates	45%
Healthy children	45–65%
Healthy adults	30–45%
Removable denture wearers	50–65%
Long term facilities	65–88%
Acute leukemia undergoing chemotherapy	90% (approximately)
HIV patients	95% (approximately)

Systemic candidiasis is less frequent but carries a mortality rate of 71–79%. The annual incidence of bloodstream infection (BSI) associated with candida ranges from 6–23/100,000 to 2.53–11/100,000 individuals in USA and European countries, respectively. Overall NCAC species have shown an increasing trend as causative pathogens in BSIs with a 10–11% increment over a 6.5-year period in a global report. In addition to *C. albicans*, the common NCAC species involved in BSIs include *C. parapsilosis* (premature neonates and catheterized patients); *C. glabrata* (elderly patients); *C. tropicalis* (hematological malignancies); and *C. krusei* (Richardson, 2005).

## Factors Predisposing for Oral Candidiasis (**Table 2**)

### Local Factors

#### Saliva

Salivary gland dysfunction predisposes to oral candidiasis. Constituents of saliva such as histidine-rich polypeptides, lactoferrin, lysozyme, and sialoperoxidase inhibit the overgrowth of candida. Hence, conditions affecting the quantity and quality of salivary secretions may lead to an increased risk of oral candidosis (Scully et al., 1994; Turner and Ship, 2007).

**Table 2:** Factors predisposing for oral candidiasis (Rautemaa and Ramage, 2011).

Local factors	Systemic factors
• Impaired local defense mechanisms	• Impaired systemic defense mechanisms
• Decreased saliva production	• Primary or secondary immunodeficiency
• Smoking	• Immunosuppressive medications
• Atrophic oral mucosa	• Endocrine disorders- Diabetes
• Mucosal diseases (Oral lichen planus)	• Malnutrition
• Topical medications – corticoids	• Malignancies
• Decreased blood supply (radiotherapy)	• Congenital conditions
• Poor oral hygiene	• Broad spectrum antibiotic therapy
• Dental prostheses	
• Altered or immature oral flora	

#### Dental Prostheses

Dental prostheses creates a favorable microenvironment for the candida organisms to thrive. Approximately 65% of complete denture wearers are predisposed to candida infection. The possible explanations include enhanced adherence of candida to the acrylic, ill-fitted appliances, decreased saliva flow under the denture surfaces or inadequate hygiene (Ashman and Farah, 2005; Martori et al., 2014).

#### Topical Medications

Another important local factor increasing the risk of oral candidosis could be use of topical or inhalational corticosteroids and overzealous use of antimicrobial mouthwashes. They temporarily suppress the local immunity and cause alterations in the oral flora (Scully et al., 1994; Jainkittivong et al., 2007).

#### Smoking

Some studies suggest that smoking alone or in combination with other factors, significantly affects the oral candida carriage while few studies propose otherwise (Soysa and Ellepola, 2005; Barnett, 2008; Munshi et al., 2015). The precise mechanism is not established but various theories have been postulated. The possible explanations facilitating candida colonization include localized epithelial alterations caused by smoking (Arendorf and Walker, 1980); smoking in association with denture friction altering the mucosal surface (Arendorf and Walker, 1987); nutritional products obtained through enzymatic breakdown of aromatic hydrocarbons contained in cigarette smoke (Hsia et al., 1981; Krogh et al., 1987); suppression of local immunity and reduction in gingival exudate; elevation of glycosylated hemoglobin levels and lastly tobacco smoke increasing the adrenaline levels in blood, indirectly affecting the blood glucose levels.

#### Diet

Unbalanced dietary intake of refined sugars, carbohydrates and dairy products (containing high content of lactose) might serve as growth enhancers by reducing the pH levels and hence favoring the candida organisms to thrive (Martins et al., 2014).

### Systemic Factors

#### Age

Extremes of age may predispose to candidiasis due to immature or weakened immunity (Weerasuriya and Snape, 2008).

#### Nutritional Status

Among the nutritional deficiency states, iron has been the most common deficient essential micronutrient implicated in the colonization of candida. Deficiency of iron diminishes the fungistatic action of transferrin and other iron-dependant enzymes. In addition, other nutrients frequently deficit in chronic candidiasis includes essential fatty acids, folic acid, vitamins A and B6, magnesium, selenium, and zinc (Paillaud et al., 2004; Martins et al., 2014).

#### Systemic Drugs

Prolonged use of systemic drugs like broad-spectrum antibiotics, immune-suppressants and drugs with xerostomic side-effects, alter the local oral flora or disrupt mucosal surface or reduce the salivary flow, creating a favorable environment for candida to grow (Martins et al., 2014). Escalation in candida organisms has also been reported in patients undergoing radiation therapy to the head and neck region.

#### Endocrine Disorders

Various reports reveal that oral and invasive candidiasis are more prevalent in patients with endocrine dysfunctions such as diabetes and Cushing’s syndrome (Graham and Tucker, 1984; Bakker et al., 1998; Sashikumar and Kannan, 2010).

#### Immune Disorders

Immunodeficiency conditions such as AIDS and severe combined immunodeficiency syndrome (SCID) are also predisposing factors for candidiasis (Anil and Challacombe, 1997; Owotade and Patel, 2014).

#### Malignancies

The host defense mechanisms are compromised by chemotherapy and radiotherapy administered for the treatment of malignant conditions. The prevalence of oral candidiasis for all cancer treatments, according to a systematic review, was reported to be 7.5% pre-treatment, 39.1% during treatment and 32.6% post-cancer therapy. The prevalence of oral candidiasis during head and neck radiation therapy and chemotherapy was observed to be 37.4 and 38%, respectively. The colonization by *C. albicans* was reported to be 46.2%. The prevalence of NCAC species were *C. tropicalis* (16.6%), *C. glabrata* (5.5%), and *C. krusei* (3%) (Scheffel et al., 2010).

#### Congenital Conditions

Lastly, individuals affected by congenital conditions associated with defective immune system such as Di George’s syndrome, hereditary myeloperoxidase deficiency and Chediak–Higashi syndrome are commonly predisposed to candida infections (Ashman and Farah, 2005).

## Forms of Oral Candida Infections (**Table 3**)

### Primary Oral Candidiasis

#### Primary Triad

##### Pseudomembranous candidiasis

**FIGURE 1:**
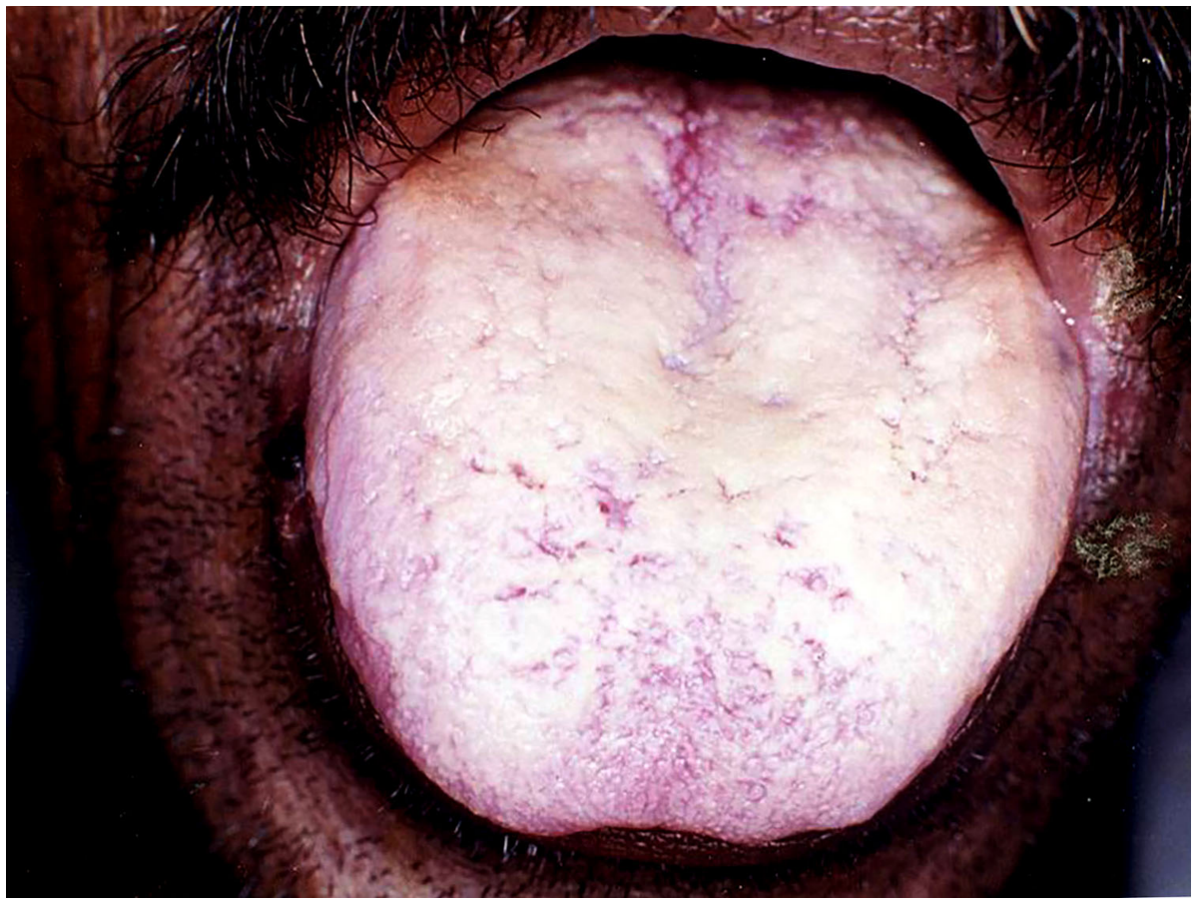
**Pseudomembranous candidiasis of the tongue.** The copyright of the images is owned by Prof. Anil and a written consent was obtained for the **Figures 1–6**.

This form of candidiasis classically presents as acute infection, though the term chronic pseudomembranous candidiasis has been used to denote chronic recurrence cases. It is commonly seen in extremes of age, immunocompromised patients especially in AIDS, diabetics, patients on corticosteroids, prolonged broad-spectrum antibiotic therapy, hematological, and other malignancies (**Figure 1**). On the oral surfaces, the superficial component presents as white to whitish-yellow creamy confluent plaques resembling milk curds or cottage cheese. These plaques consist of desquamated epithelial cells, tangled aggregates of fungal hyphae, fibrin, and necrotic material (Lalla et al., 2013). The superficial pseudo-membrane can be removed by wiping gently, leaving behind an underlying erythematous and occasionally bleeding surface (Ashman and Farah, 2005; Farah et al., 2010). The oral surfaces frequently involved include labial and buccal mucosa, tongue, hard and soft palate and oropharynx. The involvement of both oral and oesophageal mucosa is prevalent in AIDS patients. The symptoms of the acute form are rather mild and the patients may complain only of slight tingling sensation or foul taste, whereas, the chronic forms may involve the oesophageal mucosa leading to dysphagia and chest pains. Few lesions mimicking pseudomembranous candidiasis could be white coated tongue, thermal and chemical burns, lichenoid reactions, leukoplakia, secondary syphilis and diphtheria (Lalla et al., 2013).

**Table 3:** Classification of oral candidosis (Axell et al., 1997).

Primary oral candidosis	Secondary oral candidosis
**Acute forms**	**Oral manifestations of systemic mucocutaneous candidosis**
Pseudomembranous	Thymic aplasia
Erythematous	Candidosis endocrinopathy syndrome
**Chronic forms**
Hyperplastic (nodular or plaque-like) Erythematous Pseudomembranous
**Candida-associated lesions**
Denture stomatitis Angular cheilitis Median rhomboid glossitis
**Keratinized primary lesions with candidal super infection**
Leukoplakia Lichen planus Lupus erythematosus

##### Erythematous candidiasis

**FIGURE 2:**
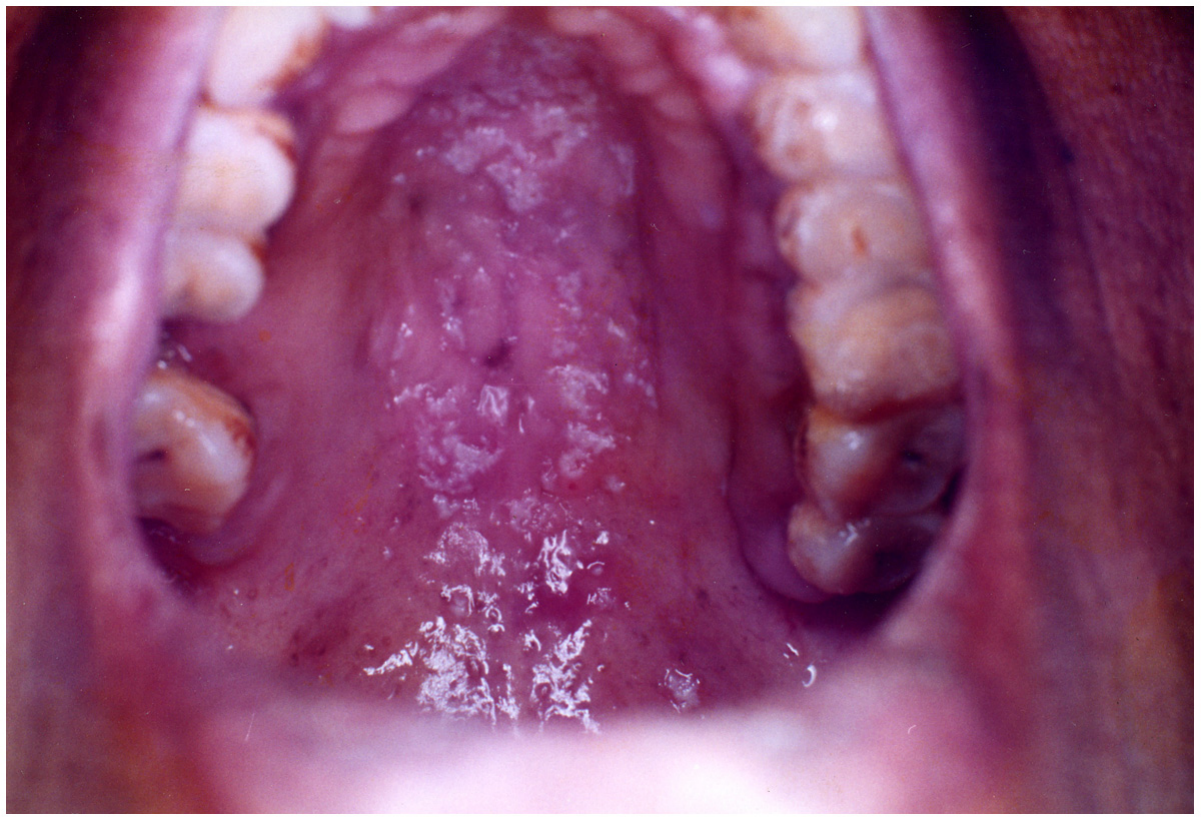
**Erythematous candidiasis of the palate**.

Erythematous candidiasis is relatively rare and manifests as both acute and chronic forms (Ashman and Farah, 2005). Previously known as ‘antibiotic sore mouth,’ due to its association with prolonged use of broad-spectrum antibiotics (Farah et al., 2010). The chronic form is usually seen in HIV patients involving the dorsum of the tongue and the palate and occasionally the buccal mucosa (**Figure 2**). Clinically, it manifests as painful localized erythematous area. It is the only form of candidiasis associated with pain. The lesions are seen on the dorsum of the tongue typically presenting as depapillated areas. Palatal lesions are more common in HIV patients. Differential diagnosis may include mucositis, denture stomatitis, erythema migrans, thermal burns, erythroplakia, and anemia (Dodd et al., 1991).

##### Hyperplastic candidiasis

**FIGURE 3:**
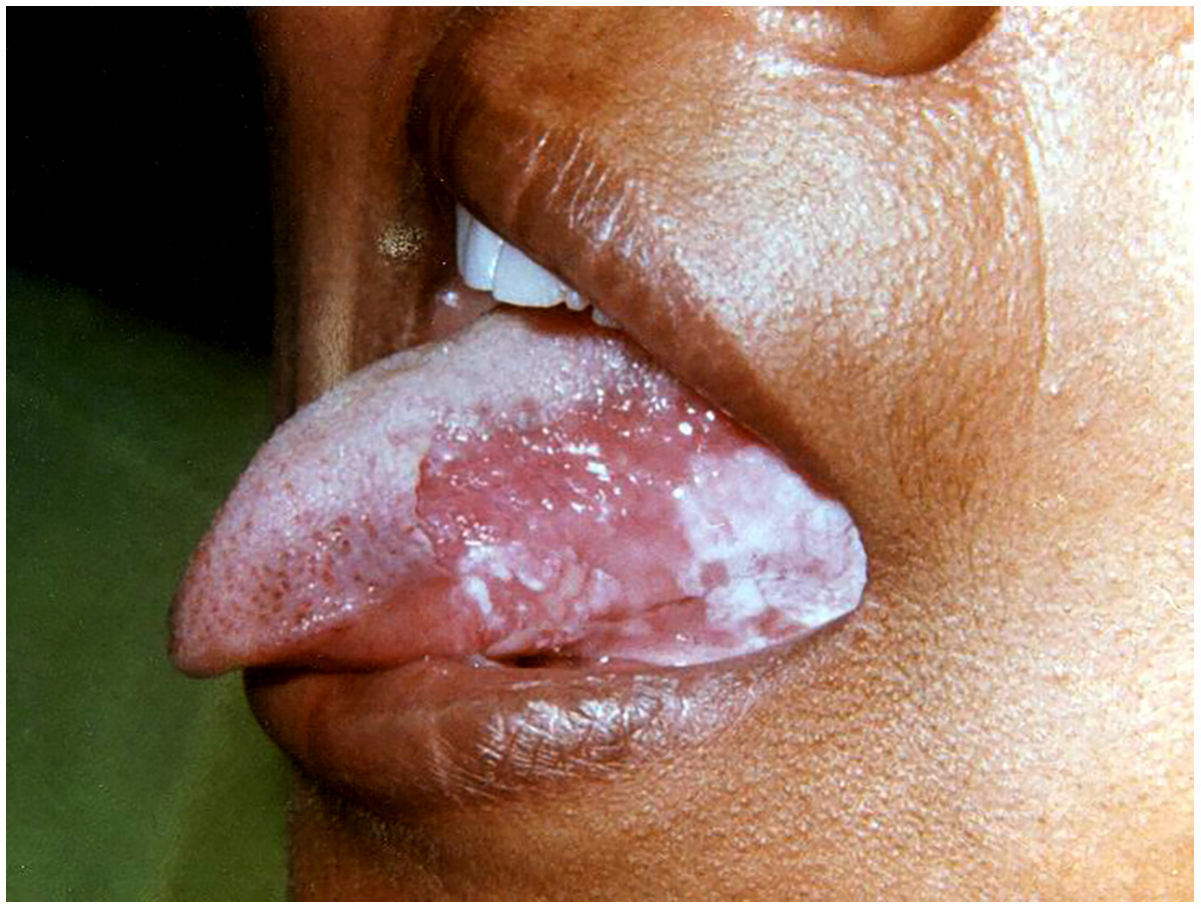
**Hyperplastic candidiasis at the lateral border of the tongue**.

The hyperplastic candidiasis mainly presents as chronic form. It has been commonly referred previously by several authors as ‘candidal leukoplakia.’ Clinically, it may manifest as one of the two variants; homogeneous adherent white plaque-like or erythematous multiple nodular/speckled type (Holmstrup and Bessermann, 1983; Sanketh et al., 2015). The lesions usually occur bilaterally in the commissural region of the buccal mucosa and less frequently on the lateral border of the tongue and palate (**Figure 3**). Unlike the pseudomembranous type, hyperplastic candidiasis lesions are non-scrapable. There appears to be a positive association with smoking and in addition may present with varying degrees of dysplasia (Williams and Lewis, 2011). A confirmed association between Candida and oral cancer is yet to be recognized, although *in vitro* studies have shown that the candida organisms can generate carcinogenic nitrosamine (Farah et al., 2010; Sanketh et al., 2015). A small percentage of cases occur in association with iron and folate deficiencies and with defective cell-mediated immunity. Differential diagnosis may include leukoplakia, lichen planus, angular cheilitis and squamous cell carcinoma.

#### Candida-associated Lesions

##### Denture stomatitis

**FIGURE 4:**
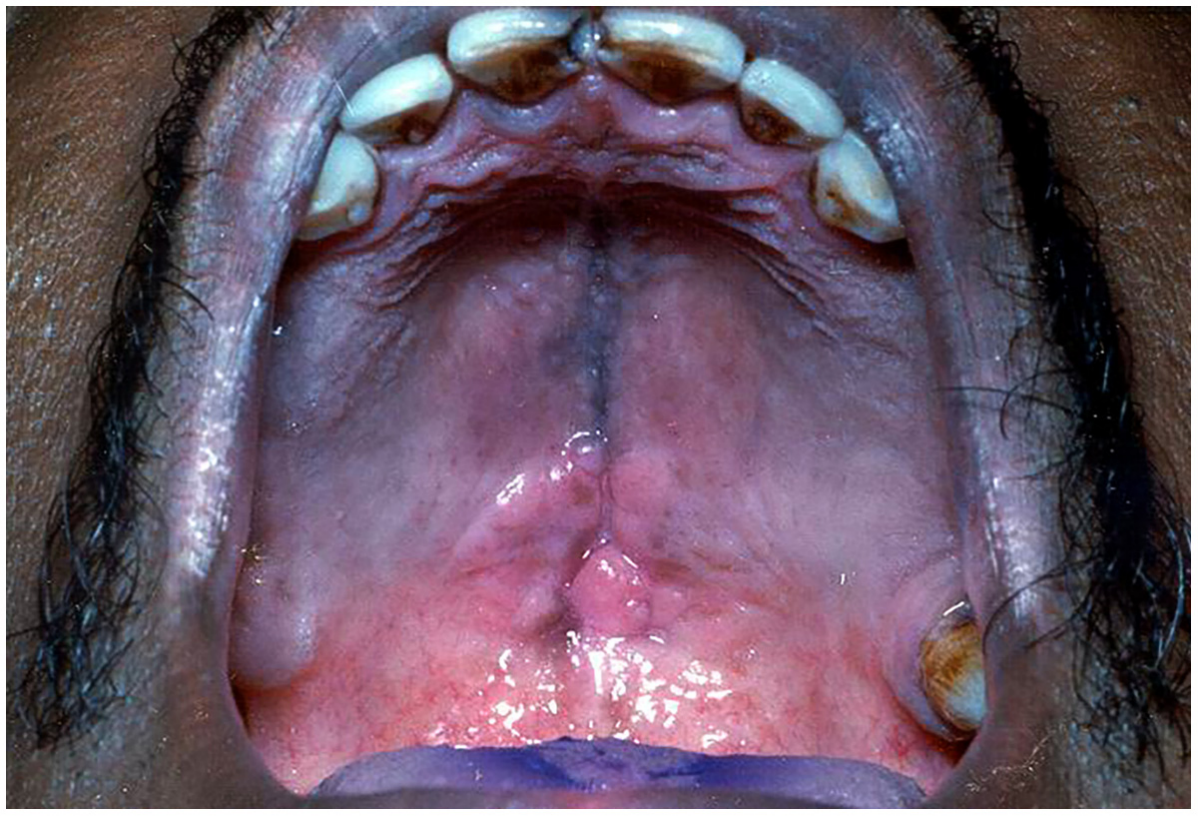
**Denture stomatitis of the palate**.

It is also known as “chronic atrophic candidiasis.” As the name indicates, it is chronic inflammation of the mucosa typically restricted to the denture-bearing area, seen in association with candidiasis (Lund et al., 2010). It is seen in almost 50–65% of the denture wearers (Ashman and Farah, 2005; Williams and Lewis, 2011). Clinically, the lesions may be seen as pinpoint hyperaemia, diffuse erythematous or granular/papillary type. It occurs frequently along with angular cheilitis and median rhomboid glossitis. The lesions are usually asymptomatic, though occasionally patients may complain of burning sensation or soreness. It commonly affects the palate although mandibular mucosa may also be affected (**Figure 4**). The associated etiological factors include poor oral hygiene practice, nocturnal denture wear, ill-fitting prostheses and limited flow of saliva (Farah et al., 2010; Williams and Lewis, 2011).

##### Angular cheilitis

This form of candidiasis usually manifests as erythematous or ulcerated fissures, typically affecting unilaterally or bilaterally the commissures of the lip (Samaranayake et al., 1995; Sharon and Fazel, 2010). Angular cheilitis often represents an opportunistic infection of fungi and/or bacteria, with multiple local and systemic predisposing factors involved in the initiation and persistence of the lesion (Park et al., 2011). The factors associated include old age and denture-wearers (due to reduced vertical dimension), vitamin B12 deficiency and iron deficiency anemia (Jenkins et al., 1977). Other causative organisms implicated are *Staphylococcus* and *Streptococcus* (Farah et al., 2010).

##### Median rhomboid glossitis

**FIGURE 5:**
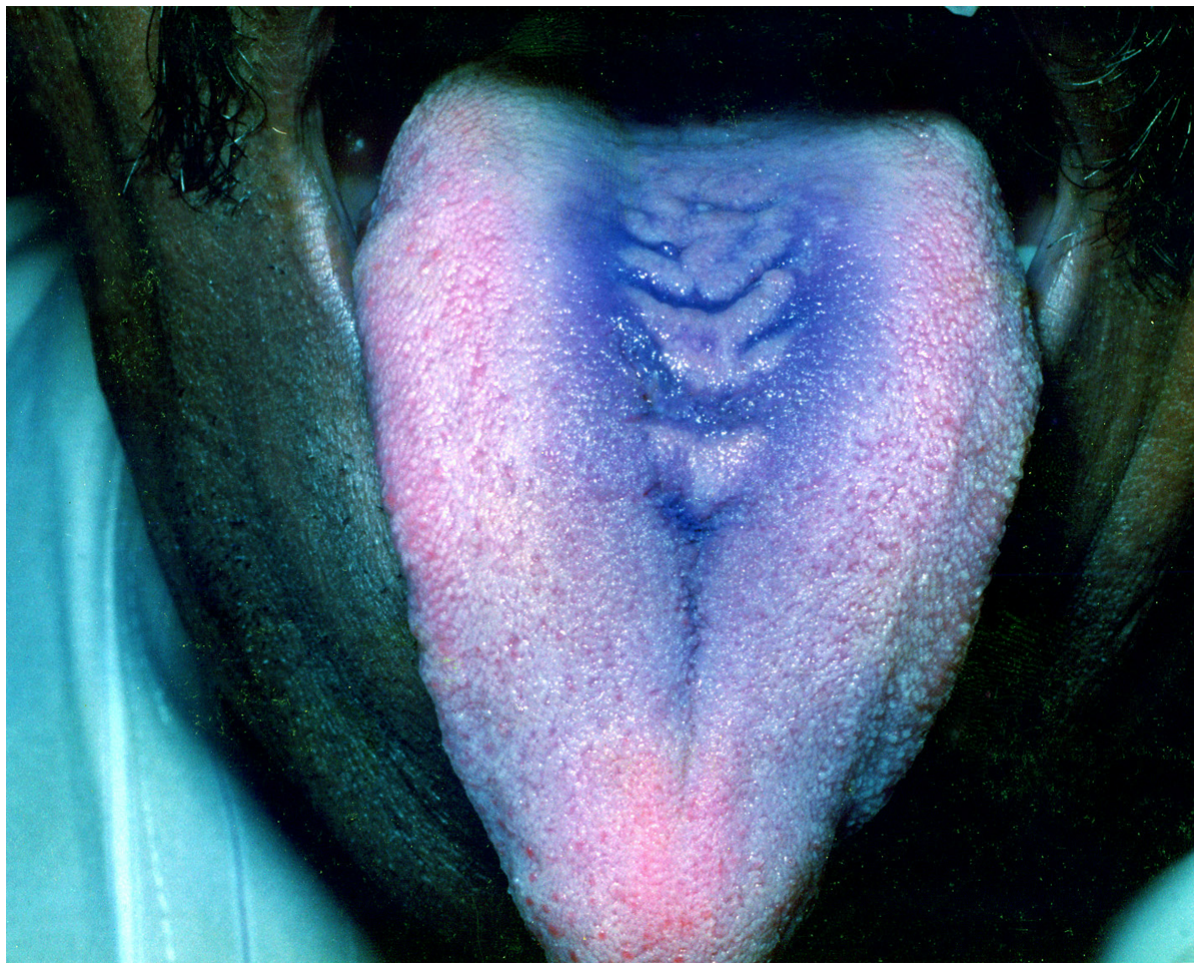
**Median Rhomboid glossitis-note the candidal overgrowth**.

Median rhomboid glossitis appears as the central papillary atrophy of the tongue and is typically located around the midline of the dorsum of the tongue. It occurs as a well-demarcated, symmetric, depapillated area arising anterior to the circumvallate papillae (**Figure 5**). The surface of the lesion can be smooth or lobulated (Joseph and Savage, 2000). While most of the cases are asymptomatic, some patients complain of persistent pain, irritation, or pruritus (Lago-Mendez et al., 2005). The lesion is now believed to be a localized chronic infection by *C. albicans*. It is commonly seen in tobacco smokers and inhalation-steroid users (Aun et al., 2009; Williams and Lewis, 2011).

##### Linear gingival erythema

**FIGURE 6:**
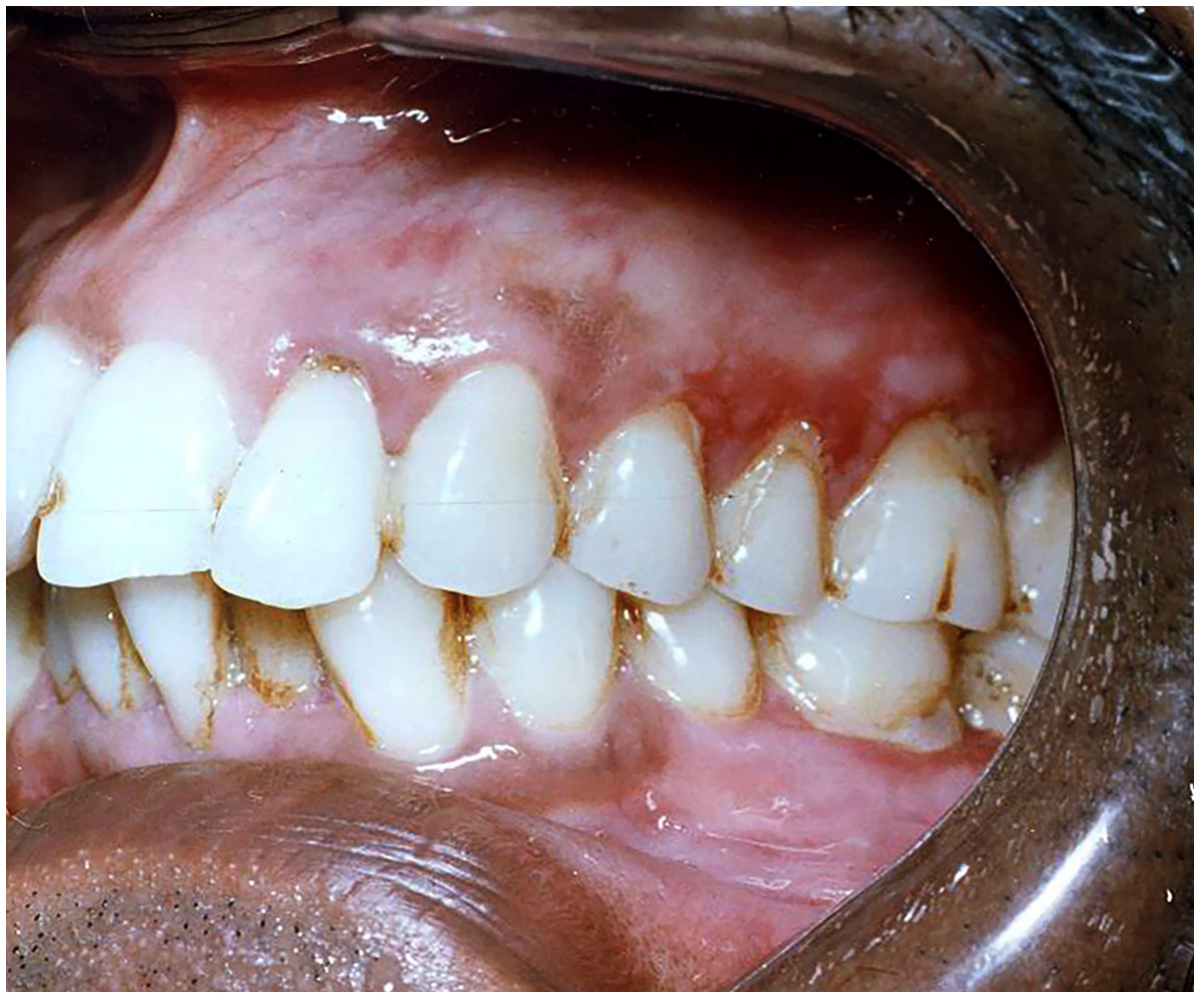
**Linear Gingival erythema in an HIV infected patient**.

It was previously referred to as “HIV-gingivitis” since its typical occurrence was in HIV associated periodontal diseases (**Figure 6**). It manifests as linear erythematous band of 2–3 mm on the marginal gingiva along with petechial or diffuse erythematous lesions on the attached gingiva. The lesions may present with bleeding. In addition to *C. albicans*, *C. dubliniensis* has been reported as an emerging pathogen in this form of candidiasis (Williams and Lewis, 2011).

### Secondary Oral Candidiasis

This group is characterized by chronic mucocutaneous candidiasis, which consists of heterogeneous disorders, presenting as persistent or recurrent superficial candida infections of the mouth, skin, nail beds, and occasionally producing granulomatous masses over the face and scalp. The primary clinical features include chronic oral, cutaneous and vulvovaginal candidiasis. Oral cavity involvement is reported in more than 90% cases and the lesions can occasionally spread into the larynx, pharynx or esophagus but further involvement is infrequent. It is associated with diverse immunodeficiency disorders such as, Di George syndrome, hyper-immunoglobulin E syndrome, Nezelof’s syndrome MPO deficiency, SCID syndrome and endocrine disorders like Addison’s disease and hypoparathyroidism (Ashman and Farah, 2005; Farah et al., 2010; Williams and Lewis, 2011; Lalla et al., 2013).

## Oral Candida Infection in Newborns

Oral candidiasis in neonates is reported to be 0.5–20%, depending upon the various studies (Yilmaz et al., 2011; Stecksen-Blicks et al., 2015). The most common form of candidiasis affecting this age group is the acute pseudomembranous candidiasis (Berdicevsky et al., 1984). *Candida* species isolated from these lesions include *C. albicans*, followed by *C. glabrata, C. tropicalis* and *C. krusei* (Tinoco-Araujo et al., 2013). Majority of the lesions are asymptomatic. They mainly present as white scrapable pseudomembranous lesions. The major predisposing factors were low birth weight, prolonged hospital stay and associated increased risk of exposure to environmental factors. The participation of dental surgeon is essential in early diagnosis of the oral signs and symptoms of this opportunistic infection in order to prevent disseminated candidiasis and subsequent mortality (2–20%; Sitheeque and Samaranayake, 2003). Treatment for superficial infection is topical administration of antifungals such as 1% clotrimazole solution thrice daily for 7 days. In case of invasive or disseminated candidiasis, systemic interventions are obligatory (Sitheeque and Samaranayake, 2003).

## Management of Oral Candidosis

An effective management of oral candidiasis can be achieved by adhering to the following simple guidelines:

(1) Diagnosis through detailed medical and dental history, clinical manifestations confirmed with laboratory tests.
(2) Correction of predisposing factors where achievable.
(3) Maintenance of proper hygiene of the oral cavity and oral prostheses, if any.
(4) Selection of antifungal therapy based on severity of the infection and susceptibility of the *Candida* species prevalent in that patient.

Diagnosis of oral candidosis, includes identification of clinical signs and symptoms, presence of the candida organisms on direct examination of a smear from the lesion or biopsy examination showing hyphae in the epithelium, positive culture, and serological tests (Rossie and Guggenheimer, 1997; Ellepola and Morrison, 2005). Another concern with respect to the treatment, is the increase in NCAC species which are naturally resistant to some of the common antifungal drugs (**Table 4**). For example, in HIV positive cases there is reported increase in *C. glabrata*, followed by *C. krusei;* in insulin using diabetes mellitus patients’ significant percentage of *C. dubliniensis* and *C. glabrata* was noted; also certain mucosal lesions, oral cancer and elderly hospitalized patients have shown increase in NCAC species carriage (Gutierrez et al., 2002).

**Table 4:** Susceptibility of *C. albicans* and common NCAC species (Gutierrez et al., 2002; Pappas et al., 2009).

*Candida* species	Fluconazole	Itraconazole	Amphotericin B	Echinocandin	Flucytosine
*Candida albicans*	S	S	S	S	S
*Candida tropicalis*	S	S	S	S	S
*Candida glabrata*	S-DD to R	S-DD to R	S-I	S	S
*Candida krusei*	R	S-DD to R	S to S-I	S	S-I to R
*Candida dubliniensis*	S to R	S to R	S	S	S

*S, Susceptible; S-DD, Susceptible dose-dependent; S-I, Susceptible intermediate; R, Resistant.*

## Antifungal Agents

**FIGURE 7:**
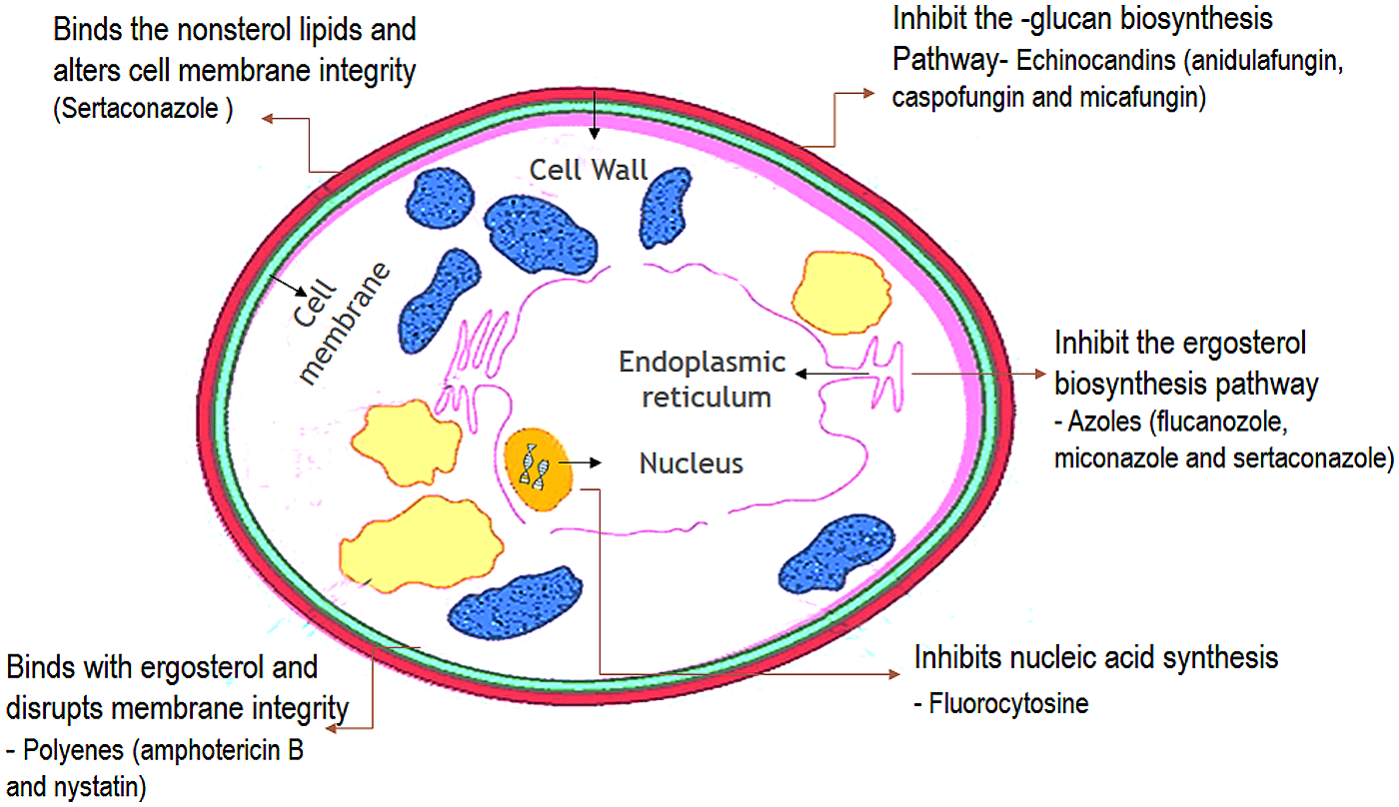
**Cellular targets of antifungal agents**. (The antifungal agents target three cellular components of fungi. Azoles inhibit the synthesis of ergosterol in the endoplasmic reticulum of the fungal cell. Polyenes such as amphotericin B bind to ergosterol in the fungal membrane causing disruption of membrane structure and function. Flucytosine is converted within the fungal cell to 5-fluorouracil which inhibits DNA synthesis.)

Antifungal agents that are available for the treatment of candidosis fall into three main categories: the polyenes (nystatin and amphotericin B); the ergosterol biosynthesis inhibitors-the azoles (miconazole, clotrimazole, ketoconazole, itraconazole, and fluconazole), allylaminesthiocarbamates, and morpholines; and DNA analog 5-fluorocytosine, and newer agents such as caspofungins (Ghannoum and Rice, 1999; Pappas et al., 2009). The choice of antifungal treatment depends on the nature of the lesion and the immunological status of the patient. There are three main antifungal drug targets in *Candida*: the cell membrane, cell wall, and nucleic acids (**Figure 7**) (Cannon et al., 2007).

Superficial oral candidosis in generally healthy patients can be treated topically and oral candidosis in immunocompromised patients should be treated systemically as well as topically. Patients with persisting risk factors and relapsing candidosis should be treated with antifungals with the lowest risk of development or selection of resistant strains (Soysa et al., 2008; Rautemaa and Ramage, 2011). The commonly used antifungal agents in the management of OPC is listed in **Table 5**.

**Table 5:** Treatment of oropharyngeal candidiasis (OPC; Thompson et al., 2010).

Severity	Antifungal drug	Dosage/ Duration
**First-line agents**		
	Fluconazole (PO or IV)	100–200 mg/7–14 days
	Clotrimazole troches	10 mg five times/7–14 days
	Nystatin suspension (100,000 U/mL)	4–6 ml four times/7–14 days
	Nystatin pastilles (200,000 U each)	1–2 pastilles four times/7–14 days
**Second-line agents**		
	Itraconazole solution (PO)	200 mg/28 days
	Posaconazole (PO)	400 mg daily in divided doses
	Voriconazole (PO or IV)	200 mg twice daily
**Agents used in refractory case of OPC**		
	Caspofungin (IV)	70 mg loading dose followed by 50 mg daily
	Micafungin (IV)	100-150 mg daily
	Anidulafungin (IV)	100 mg loading dose followed by 50 mg daily
	Amphotericin B oral suspension	500 mg every 6 h
	Amphotericin B deoxycholate (IV)	0.3 mg/kg once

## Topical Antifungals

Topical antifungals are usually the drug of choice for uncomplicated, localized candidiasis in patients with normal immune function. High levels can be achieved in the oral epithelium with topically administered antifungals. Polyenes are fungicidal drugs that act through direct binding to the ergosterol within the fungal cell membranes, inducing leakage of cytoplasmic contents leading to the fungal cell death. Nystatin or amphotericin B solutions are used for 4 weeks. In recurrent cases the duration of treatment should be for at least 4–6 weeks.

Topically administered miconazole gel is also suitable for the treatment of uncomplicated infections in generally healthy patients (Bensadoun et al., 2008). It should also be used for 1 week after resolution of symptoms. The gel inhibits the action of fungal ergosterol synthesis; interacts with the cytochrome P450 enzyme 14-alpha demethylase; inhibits growth of pathogenic yeasts by altering cell membrane permeability. Repeated use of miconazole, however, may cause a risk of development of azole-resistant strains (Rautemaa et al., 2008).

## Systemic Antifungals

Systemic antifungals are usually indicated in cases of disseminated disease and/or in immunocompromised patients. Azoles are fungistatic drugs that inhibit the fungal enzyme lanosterol demethylase responsible for the synthesis of ergosterol. Among the azoles, fluconazole attains a higher concentration in the saliva making it principally the suitable drug for treating this oral infection. Fluconazole and itraconazole are administered orally and it gets secreted onto mucous membranes. The oral solution also has a topical effect (Pappas et al., 2004). The other antifungals, echinocandins, and flucytosine act through inhibition of D-glucan synthase and DNA/protein synthesis, respectively (Muir et al., 2009; Vandeputte et al., 2012). Posaconazole, is available only as an oral solution and is used in immunocompromised patients and patients resistant to other drugs (Clark et al., 2015).

One of the risks while using fluconazole and other drugs of the azole group is the development of resistant strains (Siikala et al., 2010). For fluconazole-refractory disease, either itraconazole solution at a dosage of 200 mg daily or posaconazole suspension at a dosage of 400 mg twice daily for 3 days, then 400 mg daily for up to 28 days, are recommended. Voriconazole at a dosage of 200 mg twice daily or a 1-mL oral suspension of AmB-d, administered at a dosage of 100 mg/mL four times daily, are recommended when treatment with other agents has failed. Intravenous echinocandin or AmB-d at a dosage of 0.3 mg/kg daily can be used in treating patients with refractory disease (Vazquez, 2003).

## Alternative Anti Candidal Agents

Lastly, to mention a few natural anti-yeast substances which can be used as an alternative treatment. These agents with recognized activity against *C. albicans* includes berberine-containing plants; caprylic acid; grapefruit seed extract; garlic; probiotics; tea tree oil and enteric-coated volatile oil preparations containing cinnamon, ginger, oregano, peppermint and rosemary; propolis and thyme (Hofling et al., 2010; Valera et al., 2013). Agents capable of inhibiting microbial growth such as xylitol is known to inhibit microbial metabolism in the oral cavity. It is therefore incorporated in chewing gums and tablets as well as in health care products such as dentifrice and oral rinses. Although it has a limited effect on *Candida*, it could be beneficial in prevention of the mixed biofilm infection (Pizzo et al., 2000). The essential oil of *Melaleuca alternifolia*, also known as tea tree oil has been shown to be promising as a topical antifungal agent, with recent clinical data indicating efficacy in the treatment of oral candidiasis (Hammer et al., 2004; Sitheeque et al., 2009).

## Prevention of Oral Candidosis

Good oral hygiene practices may help to prevent oral thrush in people with weakened immune systems. Careful mechanical cleaning of teeth and dentures with a toothbrush is the cornerstone of the prevention of candida infections. Oral decontamination using antifungal and antibacterial rinses is one of the approaches often used to manage oral mucositis (Fathilah et al., 2012). Chlorhexidine digluconate, and cetylpyridinium chloride are two antiseptics often incorporated in mouth rinses and used as prophylaxis for both chemotherapy and radiotherapy induced mucositis (Salim et al., 2013). People who use inhaled corticosteroids may be able to reduce the risk of developing thrush by washing out the mouth with water or mouthwash after using an inhaler (Soares et al., 2011). For susceptible denture wearers, it is advisable to remove the denture at night and soak in 0.2% Chlorhexidine solution or 15–30 min in white vinegar (diluted 1:20) or 0.1% hypochlorite solution (Kassaify et al., 2008). The elimination or at least regulation of the predisposing factors for candidiasis is essential. Failure to recognize this may only provide a temporary relief using antifungal therapy, but with inevitable relapse of the infection (Akpan and Morgan, 2002).

## Conclusion

In the past few decades extensive clinical data has been recorded on oral candidiasis with respect to its advent with the various immunocompromised conditions. With the increasing incidence of NCAC species and the development of antifungal resistance, there is a persistent requirement in research for newer effective agents. One such prospect is development of vaccine against candida organisms. Various experimental strategies have been employed for developing such a vaccine, like attenuated live candida organisms, SAP gene family proteins, glycoconjugates (mannans and β-glucans) to mention a few, but clinical trials are still a distant vision.

## Conflict of Interest Statement

The authors declare that the research was conducted in the absence of any commercial or financial relationships that could be construed as a potential conflict of interest.
